# Molecular epidemiology of *Plasmodium vivax* and *Plasmodium falciparum* malaria among Duffy-positive and Duffy-negative populations in Ethiopia

**DOI:** 10.1186/s12936-015-0596-4

**Published:** 2015-02-19

**Authors:** Eugenia Lo, Delenasaw Yewhalaw, Daibin Zhong, Endalew Zemene, Teshome Degefa, Kora Tushune, Margaret Ha, Ming-Chieh Lee, Anthony A James, Guiyun Yan

**Affiliations:** Program in Public Health, College of Health Sciences, University of California at Irvine, Irvine, CA 92697 USA; Department of Medical Laboratory Sciences and Pathology, College of Public Health and Medical Sciences, Jimma University, Jimma, Ethiopia; Department of Health Services Management, College of Public Health and Medical Sciences, Jimma University, Jimma, Ethiopia; Departments of Microbiology & Molecular Genetics and Molecular Biology & Biochemistry, University of California at Irvine, Irvine, CA 92697 USA

**Keywords:** Age distribution, Geographical distribution, Human *DARC* gene, Quantitative PCR, Parasite gene copy number

## Abstract

**Background:**

Malaria is the most prevalent communicable disease in Ethiopia, with 75% of the country’s landmass classified as endemic for malaria. Accurate information on the distribution and clinical prevalence of *Plasmodium vivax* and *Plasmodium falciparum* malaria in endemic areas, as well as in Duffy-negative populations, is essential to develop integrated control strategies.

**Methods:**

A total of 390 and 416 community and clinical samples, respectively, representing different localities and age groups across Ethiopia were examined. Malaria prevalence was estimated using nested PCR of the 18S rRNA region. Parasite gene copy number was measured by quantitative real-time PCR and compared between symptomatic and asymptomatic samples, as well as between children/adolescents and adults from the local community. An approximately 500-bp segment of the human *DARC* gene was amplified and sequenced to identify Duffy genotype at the -33rd nucleotide position for all the clinical and community samples.

**Results:**

*Plasmodium vivax* prevalence was higher in the south while *P. falciparum* was higher in the north. The prevalence of *P. vivax* and *P. falciparum* malaria is the highest in children compared to adolescents and adults. Four *P. vivax* infections were detected among the Duffy-negative samples. Samples from asymptomatic individuals show a significantly lower parasite gene copy number than those from symptomatic infections for *P. vivax* and *P. falciparum*.

**Conclusions:**

Geographical and age differences influence the distribution of *P. vivax* and *P. falciparum* malaria in Ethiopia. These findings offer evidence-based guidelines in targeting malaria control efforts in the country.

**Electronic supplementary material:**

The online version of this article (doi:10.1186/s12936-015-0596-4) contains supplementary material, which is available to authorized users.

## Background

Malaria is the most prevalent communicable disease in Ethiopia, with 75% of the country’s landmass classified as malaria-endemic [1]. This disease has caused tremendous human suffering and major negative effects on economic productivity. From 2007 to 2008, malaria accounted for 10% of all hospital admissions and for ~15% of the overall disability adjusted life years (DALYs) lost in the country [1,2]. The malaria morbidity reported by the Ethiopian Government and World Health Organization (WHO) may underestimate the actual burden due to the lack of epidemiological data, in addition to poor health infrastructure and accessibility in the country [3,4]. The problem is compounded by the presence of multiple malaria parasite species [4,5], increasing drug resistance in the parasites [6,7] and insecticide resistance in the mosquito vectors [8,9]. Across the country, *Plasmodium falciparum* and *Plasmodium vivax* account for approximately 60 and 40%, respectively, of infected cases [3-5]. Nonetheless, information on epidemiological significance, i.e., the distribution and clinical prevalence of *P. falciparum* and *P. vivax* malaria in endemic areas is still insufficient.

Natural selection in malaria-endemic regions may have favoured individuals who lack the Duffy blood group antigen on the surface of their red blood cells because of the conferred resistance to certain malaria parasites [10-13]. The Duffy antigen receptor for chemokines (*DARC*), also known as Fy glycoprotein, belongs to a family of silent heptahelical chemokine receptors [10]. *Plasmodium vivax* and *Plasmodium knowlesi* require this protein to infect red blood cells during their asexual blood stage, while *P. falciparum* uses a different set of receptors to gain access to the cell [14,15]. A point mutation, T-33C, located in a GATA-1 transcription factor-binding site of the *DARC* gene promoter can lead to failure of Duffy antigen expression on the surface of red blood cells in humans [10]. The absence of a receptor for the pathogen confers resistance to *P. vivax* malaria [10,16]. The rare presence of *P. vivax* malaria in western or central Africa is likely attributed to high Duffy-negativity among African blacks (88-100%) [17,18]. However, this interpretation is challenged by recent findings of *P. vivax* infection in Duffy-negative people in different parts of Africa [19-24] and the Brazilian Amazon region [25,26]. These data support the hypothesis that *P. vivax* may have evolved the capability to infect Duffy-negative red blood cells and that the parasites are more prevalent and widespread than reported previously.

There has been a number of population-based studies of *P. vivax* infections in Duffy-negative individuals among clinical and community samples [19-21,23-27]. Accurate information on the distribution and clinical prevalence of *P. vivax* and *P. falciparum* malaria in endemic areas, as well as in Duffy-negative populations, is essential to develop integrated control strategies and to more broadly evaluate the magnitude of the ‘derived’ *P. vivax* invasion. The present study defines the epidemiology of *P. vivax* and *P. falciparum* malaria in large areas of Ethiopia with three specific questions: (1) whether there are variations in the geographical distribution of *P. vivax* and *P. falciparum* malaria across Ethiopia; (2) is there a difference in the prevalence of *P. vivax* and *P. falciparum* malaria between age groups in local communities; and, (3) what is the frequency of *P. vivax* infection in the Duffy-negative populations? Furthermore, the parasite gene copy number between symptomatic and asymptomatic infections of *P. vivax* and *P. falciparum* were compared with the goal to evaluate the performance of a quantitative real-time PCR (qPCR) method for detecting high and low parasite density samples. This is of key relevance in providing accurate epidemiological data in local communities with mostly asymptomatic infections.

## Methods

### Ethics statement

Scientific and ethical clearance was obtained from the institutional scientific and ethical review boards of Jimma University, Ethiopia and University of California, Irvine, USA. Written informed consent/assent for study participation was obtained from all consenting heads of households, parents/guardians (for minors under age of 18), and each individual who was willing to participate in the study.

### Areas of study and sample collection

**Figure 1:**
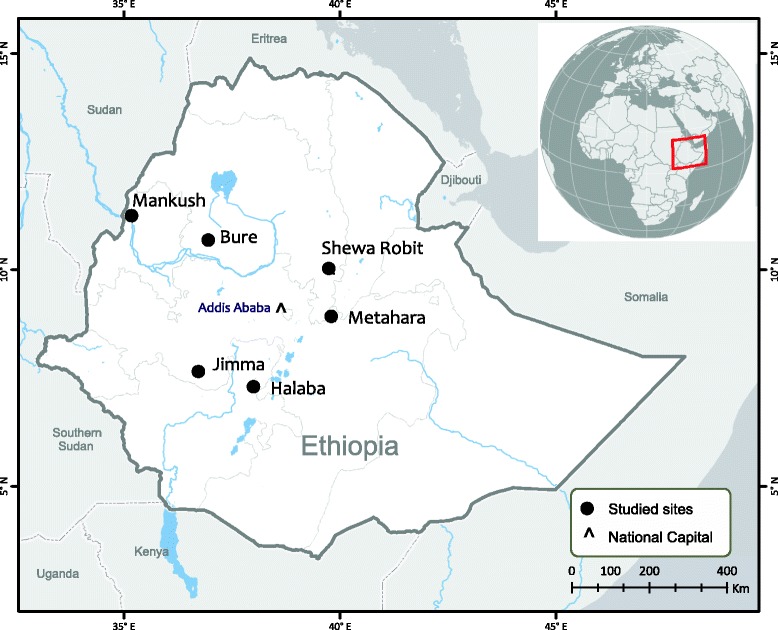
**Map showing the geographical distribution of the sample sites in Ethiopia.** Community samples were collected in Asendabo and clinical samples were collected in the other six sites.

Clinical and community samples from six different localities across Ethiopia were collected to determine malaria prevalence (Figure 1). Finger-prick blood samples were collected from a total of 416 malaria symptomatic or febrile patients visiting the health centres or hospitals at each of the localities (ranging from 41–125 patients per locality; Table 1). In addition, blood samples were collected from 390 asymptomatic individuals representing the younger age group, children and adolescents of age under 18 (*n* = 200), and the older age group, adults of age 18 or above (*n* = 190) from local communities of the Asendabo town (Figure 1). A total of three to four spots of blood, equivalent to ~50 μl, from each individual were blotted on Whatman 3MM filter paper. Parasite DNA was extracted from dried blood spots by the Saponin/Chelex method [28] and genomic DNA was eluted in a total volume of 200 μl TE buffer.

**Table 1:** **Distribution and prevalence of** ***Plasmodium vivax*** **(Pv) and** ***Plasmodium falciparum*** **(Pf) infections among the clinical samples collected from the six health-centres/hospitals across Ethiopia**

**Locality**	**Duffy phenotype**	**Number of samples**	**Total infection**	**Number of Pv infection**	**Number of Pf infection**	**Number of Pv and Pf infection**
Bure						
	Positive	54	52 (96.3%)	30 (55.6%)	14 (25.9%)	8 (14.8%)
	Negative	7	4 (57.1%)	0 (0%)	4 (57.1%)	0 (0%)
Halaba						
	Positive	66	17 (25.7%)	13 (19.7%)	2 (3%)	2 (3%)
	Negative	20	1 (5%)	0 (0%)	1 (5%)	0 (0%)
Jimma						
	Positive	117	117 (100%)	93 (79.5%)	13 (11.1%)	11 (9.4%)
	Negative	8	7 (87.5%)	0 (0%)	6 (75%)	**1 (12.5**%**)**
Mankush						
	Positive	29	26 (89.7%)	7 (24.1%)	18 (62.1%)	1 (3.4%)
	Negative	21	16 (76.2%)	0 (0%)	15 (71.4%)	**1 (4.8**%**)**
Metehara						
	Positive	33	33 (100%)	14 (42.4%)	14 (42.4%)	5 (15.2%)
	Negative	20	18 (90%)	0 (0%)	18 (90%)	0 (0%)
Shewa Robit						
	Positive	23	23 (100%)	7 (30.4%)	12 (52.2%)	4 (17.4%)
	Negative	18	17 (94.4%)	0 (0%)	17 (94.4%)	0 (0%)
**Total**		416	331	164	134	33

Numbers in bold denote the number of individuals infected with *P. vivax* and have the Duffy-negative phenotype.

### *Plasmodium* species identification and quantification

A nested amplification of the 18S rRNA gene of *Plasmodium* was performed to identify positive infection and parasite species using published protocols [29,30]. Genomic DNA of each sample was amplified in duplicate for verification. Amplification was conducted in a 20 μl reaction mixture containing 2 μl of genomic DNA, 10 μl of 2 × DreamTaq^TM^ Green PCR Master Mix (Fermentas) and 0.3 uM primer. Amplification reactions were performed in a BIORAD MyCycler thermal cycler, with an initial denaturation at 94°C for 2 min, followed by 35 cycles at 94°C for 30 sec, 58°C for 30 sec and 65°C for 40 sec, with a final 2-min extension at 65°C. A secondary amplification was conducted in a 20 μl reaction mixture containing 2 μl of product from the primary reaction and the same PCR reagents (with the exception of forward and reverse primers) and volume described above. The reactions were performed with an initial denaturation at 94°C for 2 min, followed by 35 cycles at 94°C for 20 sec, 58°C for 20 sec and 65°C for 30 sec, with a final 2-min extension at 65°C. The amplified products were resolved electrophoretically on a 2.5% agarose gel in 0.5 × Tris-acetate buffer and visualized under UV light.

Parasite gene copy number was estimated using qPCR, specifically the SYBR Green detection method [31] using modified primers (forward: 5′-AGTCATCTTTCGAGGTGACTTTTAGATTGCT-3′; reverse: 5′GCCGCAAGCTCCACGCCTGGTGGTGC-3′) specific to *P. falciparum* and (forward: 5′-GAATTTTCTCTTCGGAGTTTATTCTTAGATTGC-3′; reverse: 5′GCCGCAAGCTCCACGCCTGGTGGTGC-3′) specific to *P. vivax* that targeted the 18S rRNA genes. Based on the microscopic data, no *P. malariae* or *P. ovale* was detected in the samples of the present study. Therefore, the qPCR assay was performed to detect and quantity *P. falciparum* and *P. vivax* only. Amplification was conducted in a 20 μl reaction mixture containing 2 μl of genomic DNA, 10 μl 2 × SYBR Green qPCR Master Mix (Thermo Scientific), and 0.5 uM primer. The reactions were performed in CFX96 Touch^TM^ Real-Time PCR Detection System (BIORAD), with an initial denaturation at 95°C for 3 min, followed by 45 cycles at 94°C for 30 sec, 55°C for 30 sec, and 68°C for 1 min with a final 95°C for 10 sec. This was followed by a melting curve step of temperature ranging from 65°C to 95°C with 0.5°C increments to determine the melting temperature of each amplified product. Each assay included positive controls of both *P. falciparum* 7G8 (MRA-926) and HB3 (MRA-155) isolates as well as *P. vivax* Pakchong (MRA-342G) and Nicaragua (MRA-340G) isolates, in addition to negative controls, including uninfected samples and water. A standard curve was produced from a ten-fold dilution series of the control plasmids (*P. falciparum and P. vivax*) and laboratory culture (*P. falciparum)* ranging from 1 to 1.75 × 10^−12^% parasitaemia to determine the efficiency and detection limit of the qPCR. Melting curve analyses were performed for each amplified sample to confirm specific amplifications of the target sequence. The slope of the linear regression of threshold cycle number (*Ct*) *versus* log_10_ (gene copy number) was used to calculate amplification efficiency of each plate run based on internal standard controls. For the measure of reproducibility of the threshold cycle number, the mean *Ct* value and standard error was calculated from three independent assays of each sample. A cut-off threshold of 0.02 fluorescence units that robustly represented the threshold cycle at the log-linear phase of the amplification and above the background noise was set to determine *Ct* value for each assay. Samples yielding *Ct* values higher than 40 (as indicated in the negative controls) were considered negative for *Plasmodium* species. The amount of parasite density in a sample was quantified by converting the threshold cycle (*Ct*) into gene copy number (GCN) using the follow equation: GCN_sample_ = 2 ^E×(40-*Ct*sample)^; where GCN stands for gene copy number, *Ct* for the threshold cycle of the sample, and E for amplification efficiency. Mean and standard deviation values of the log-transformed parasite GCN of samples from each of the sites were reported. The differences in the log-transformed parasite GCN between age groups as well as between symptomatic and asymptomatic samples were assessed for significance at the level of 0.05 by one-tailed t-tests. Variations in GCN among samples were presented as boxplots showing the median and interquartile range values. For the latter comparison, only the symptomatic samples from Jimma and asymptomatic samples from Asendabo were included because these two sites are in the same area so that difference in parasite GCN would not be affected by geographical difference.

### *DARC* gene sequencing

An ~500-bp fragment of the human *DARC* gene that encompasses the -33rd nucleotide position located in the promoter region was amplified and sequenced using published protocols [11]. Amplifications were conducted in a 20 μl reaction mixture containing 2 μl of genomic DNA, 10 μl of 2 × DreamTaq^TM^ Green PCR Master Mix (Fermentas) and 0.3 uM primer. The reactions were performed in a BIORAD MyCycler thermal cycler, with an initial denaturation at 94°C for 2 min, followed by 35 cycles at 94°C for 30 sec, 58°C for 30 sec, and 65°C for 40 sec, with a final 2-min extension at 65°C in the primary amplification. Amplicons were purified with USB ExoSAP-IT (Affymetrix) followed by direct sequencing with the forward and reverse primers using the BigDye Terminator v3.1 cycle sequencing kit (Applied Biosystems) on a 3130*xl* DNA analyzer (Applied Biosystems) according to manufacturer’s instructions.

## Results

### Distribution and frequency of *Plasmodium vivax* and *Plasmodium falciparum* infections among the Duffy-positive and -negative individuals

A total of 94 of the 416 (~23%) clinical samples collected from the six health centres/hospitals were homozygous for the CC genotype at the -33rd nucleotide position (indicative of Duffy-negative). One-hundred-and-eight (~26%) were homozygous TT, and 214 (~51%) were heterozygous CT yielding 322 (~77%) Duffy-positive samples. Two of the 94 Duffy-negative samples were positive for *P. vivax*, one from the hospital in Jimma and the other one in Mankush (number in bold; Table 1). Based on the nested and quantitative PCR assays, these two infected samples were mixed infections that contain both *P. vivax* and *P. falciparum*. Compared to *P. vivax* infection, the prevalence of *P. falciparum* infection among the Duffy-negative samples was considerably higher (an average prevalence of 41%; Table 1), with the lowest prevalence observed from the clinical samples in Halaba (5%) and the highest prevalence in Shewa Robit (94%).

Three of the six health centres (Bure, Halaba and Jimma) showed a higher prevalence of *P. vivax* than *P. falciparum* infection for the Duffy-positive samples (Table 1). In Jimma, the prevalence of *P. vivax* infection was ≤ five-fold higher than that of *P. falciparum*. By contrast, the prevalence of *P. falciparum* was ≤ three-fold higher than that of *P. vivax* in Mankush and Shewa Robit (Table 1). In Metehara, an equal number of *P. vivax* and *P. falciparum-*infected cases was detected among the Duffy-positives. A total of 31 (11.6%) of the 268 infected cases in the Duffy-positives were mixed infections of both *P. vivax* and *P. falciparum*.

Among the community samples, 139 out of 390 (35.6%) were shown to be Duffy-negative and this proportion was significantly higher than the clinical samples (*χ*^2^ = 16.67, d.f. = 1, *P* < 0.0001; Table 2). The proportion of Duffy-negativity was comparable among children (24/72; 33.3%), adolescents (52/128; 40.6%), and adults (63/190; 33%; Tables 2). In general, the prevalence of *P. falciparum* was higher than that of *P. vivax* in both the Duffy-positive and Duffy-negative samples, with the exception of the Duffy-positive adolescents (aged 6–18) that showed a higher *P. vivax* (9.2%) than *P. falciparum* (3.9%) prevalence (Table 2)*.* None of the 24 Duffy-negative children (aged 0–5) were detected with *P. vivax* or *P. falciparum* infections (Table 2). Among the 52 Duffy-negative adolescents, one was infected by *P. vivax* (1.9%) and six were infected by *P. falciparum* (11.5%). No mixed infections were detected among these samples. Likewise, in adults, one of the 63 Duffy-negative samples was infected by *P. vivax* (1.6%) and ten were infected by *P. falciparum* (15.9%; Table 2). No mixed infections were detected.

**Table 2:** **Prevalence of** ***Plasmodium vivax*** **(Pv) and** ***Plasmodium falciparum*** **(Pf) asymptomatic malaria among the community samples collected in Asendabo, Ethiopia**

**Age group**		**Number of samples**	**Total infection**	**Number of Pv infection**	**Number of Pf infection**	**Number of Pv and Pf infection**
0-5 years old						
	Duffy-positive	48	23 (47.9%)	10 (20.8%)	13 (27.1%)	0
	Duffy-negative	24	0	0	0	0
	Total	72	23 (31.9%)	10 (13.9%)	13 (18.1%)	0
6-18 years old						
	Duffy-positive	76	11 (14.5%)	7 (9.2%)	3 (3.9%)	1 (1.3%)
	Duffy-negative	52	7 (13.5%)	**1 (1.9**%**)**	6 (11.5%)	0
	Total	128	18 (14.1%)	8 (6.3%)	9 (7%)	1 (0.8%)
Above 18 years old						
	Duffy-positive	127	21 (16.5%)	4 (3.1%)	17 (13.4%)	0
	Duffy-negative	63	11 (17.5%)	**1 (1.6**%**)**	10 (15.9%)	0
	Total	190	32 (16.8%)	5 (2.6%)	27 (14.2%)	0
Combined all age groups						
	Duffy-positive	251	55 (21.9%)	21 (8.4%)	33 (13.1%)	1 (0.4%)
	Duffy-negative	139	18 (12.9%)	**2 (1.4**%**)**	16 (11.5%)	0
	Total	390	73 (18.7%)	23 (5.9%)	49 (12.6%)	1 (0.3%)

Numbers in bold denote the number of individuals infected with *P. vivax* and have the Duffy-negative phenotype.

### Infection prevalence and parasite gene copy number among age groups

**Figure 2:**
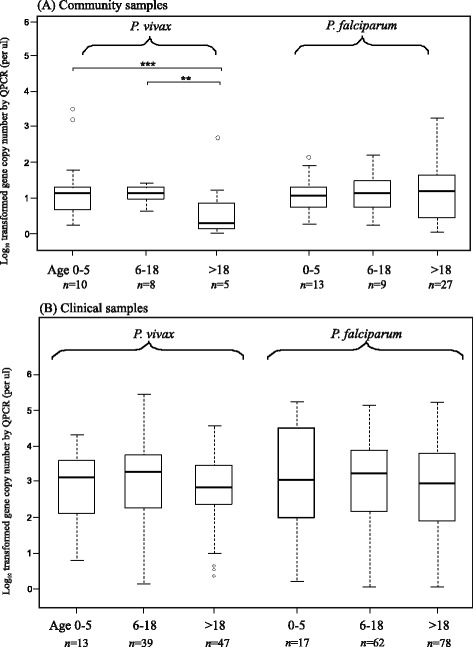
**Box plots of the log-transformed parasite gene copy number of the community and clinical samples. (A)** Box plot of the log-transformed parasite gene copy number of *Plasmodium vivax* and *Plasmodium falciparum* measured by qPCR in children/adolescents of age under 18 and adults of age 18 and above. These samples represent the local community of Asendabo, Ethiopia. **(B)** Box plot showing the log-transformed parasite gene copy number of clinical *Plasmodium vivax* and *Plasmodium falciparum* cases in children/adolescents and adults from the six health centre/hospital sites in Ethiopia. The central box represents the interquartile range and the vertical lines represent the first and fourth quartiles of the data. The median is shown as a line through the centre of the box. Outlier samples are represented by open circles. *P*-values (above) are provided for the comparison of gene copy number between the two age groups with respect to *P. vivax* and *P. falciparum.* Numbers (bottom) indicate the number of individuals included.

The overall parasite prevalence in children aged from 0–5 (31.9%) was the highest among the three age groups in the community samples (adolescents: 14.1%; adults: 15.8%; Table 2). The prevalence of *P. vivax* infections in children (13.9%) was > two-times higher than in adolescents (6.3%) and > five-times higher than in adults (2.6%; Table 2). Among the *P. falciparum* infected samples, prevalence was the highest in children (18.1%) followed by adults (14.2%) and adolescents (7%; Table 2). Despite the small sample size of *P. vivax* infection detected in the community samples (Table 2), the *P. vivax* gene copy number in children (mean GCN and standard deviation: 1.51 ± 1.23/μl) and adolescents (1.08 ± 0.24/μl) were significantly higher than that in adults (0.49 ± 0.80/μl) (*P* < 0.05; Figure 2A). For *P. falciparum*, no significant difference was found in the parasite GCN among the three age groups (Figure 2A). Among the infected community samples, only one individual (from the adolescent aged 6–18) was found to contain both *P. vivax* and *P. falciparum* (Table 2). The proportions of *P. falciparum* infection were slightly higher than *P. vivax* in both the children and adolescents; and this difference was substantial in adults where the majority of the infections are *P. falciparum.*

Compared to the community samples, the clinical samples had a relatively small sample size for the three age groups from each of the sites (Additional file 1). This precluded any meaningful comparison of infection rate among age groups. When all clinical samples were combined, children (34.2%) showed a slightly higher prevalence of *P. vivax* compared to adolescents (32.8%) and adults (28.7%). Nonetheless, the prevalence of *P. falciparum* was similar among the three age groups. Both the *P. vivax* (children: 2.86 ± 1.01/μl; adolescents: 2.86 ± 1.22/μl; and adults: 2.80 ± 1.00/μl) and *P. falciparum* GCN (children: 3.14 ± 1.54/μl; adolescents: 3.16 ± 1.15/μl; and adults: 2.92 ± 1.11/μl) were not significantly different among the three age groups in the clinical samples (Figure 2B).

### Comparisons of parasite gene copy number between symptomatic and asymptomatic infections

**Figure 3:**
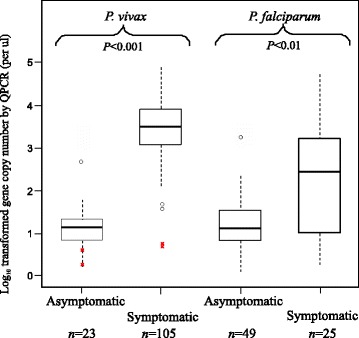
**Box plot of the log-transformed parasite gene copy number of** ***Plasmodium vivax*** **and** ***Plasmodium falciparum*** **measured by qPCR in asymptomatic and symptomatic samples from Asendabo (community) and Jimma (hospital), respectively.** The central box represents the interquartile range and the vertical lines represent the first and fourth quartiles of the data. The median is shown as a line through the centre of the box. Outlier samples are represented by open circles. The gene copy number of *P. vivax* detected in the four Duffy-negative individuals are indicated by crosses in red. Numbers (top) indicate the number of individuals included. *P*-values (below) are provided for the comparison of gene copy number between asymptomatic and symptomatic samples with respect to *P. vivax* and *P. falciparum.*

qPCR analyses revealed a significant difference in the parasite GCN among the asymptomatic (community) and symptomatic (hospital) infections for both *P. vivax* and *P. falciparum* (Figure 3). Symptomatic *P. vivax* infections showed a mean GCN of 3.45 ± 0.69/μl, which was significantly higher than that observed in the asymptomatic *P. vivax* infections (mean GCN of 1.30 ± 0.82/μl; Figure 3). Similarly, symptomatic *P. falciparum* infections showed a mean GCN of 2.48 ± 1.29/μl, which was higher than that in the asymptomatic *P. falciparum* infections (mean GCN 1.13 ± 0.41/μl; Figure 3). No significant differences were detected for the *P. vivax* and *P. falciparum* GCNs in the symptomatic samples derived from the six sites across Ethiopia (Additional file 2).

A total of four Duffy-negatives samples derived from both the community (site Asendabo) and clinic (sites Jimma and Mankush) were positive for *P. vivax* infections (Tables 1 and 2). These samples have a mean GCN of 0.66 ± 0.64/μl (Figure 3), well at the lower limit of the range of *P. vivax* GCN observed in Duffy-positive samples (a mean GCN of 3.04 ± 1.06/μl). The substantial difference in size, *n* = 4 and *n* = 210, of *P. vivax* infections in Duffy-negative and -positive samples, respectively, precludes any meaningful test of significance of the GCN. For *P. falciparum*, the comparison of parasite GCN indicated no significant difference between Duffy-positive and Duffy-negative individuals regardless of whether samples from all sites were analysed together or separately (*P* > 0.05).

## Discussion

Approximately 35% (139/390) and 22% (94/416) of the community and clinical samples analysed in this study are negative for the Duffy antigen. The proportion of Duffy-negatives observed in the clinical samples is similar to an earlier study that reported an average of 20% homozygous Duffy-negative patients in Harar and Jimma [23]. In contrast, the proportion of Duffy-negatives observed in the community is over one-third more than that in the clinical samples. However, these findings are still lower than the proportion of Duffy-negatives documented in West and Central Africa (>97%) [17,18]. Four *P. vivax* infections were identified among the Duffy-negative samples, one of which was from Jimma, one from Mankush, and the other two from Asendabo. Two of these infected samples were obtained from individuals with malaria symptoms and had mixed infections; and qPCR showed that all these samples had a relatively low number of parasite gene copy. These findings confirm previous studies that documented a number of *P. vivax* infections in Duffy-negative individuals in Cameroon [19], Madagascar [20], Angola [21], Equatorial Guinea [21], Ethiopia [23], Mauritania [24], as well as the Brazilian Amazon region [25,26]. Although these studies collectively are consistent with the conclusion that Duffy-negative individuals are not completely resistance to *P. vivax* infection, the observation of low *P. vivax* GCN in the present Duffy-negative samples supports the hypotheses that the infectivity of the parasite to human erythrocyte is reduced in the absence of the Duffy antigen. It is worth mentioned that the identification of Duffy phenotypes was inferred solely based on the *DARC* genotypes but without direct measure of antigen expression phenotypes of the erythrocytes. Thus it is not entirely impossible for a Duffy receptor to be present on the erythrocyte surfaces of a genotypically Duffy-negative individual and that the *P. vivax* strains use such to invade the erythrocytes of Duffy negatives in this study.

The mechanism of *P. vivax* erythrocyte invasion in Duffy-negatives is not yet fully understood [11,13,32]. Apart from the Duffy antigen, there are several tryptophan-rich antigens that play important role to the survival and growth of malarial parasites in the host [33-35]. For example, one of the *P. vivax* tryptophan-rich antigens PvTRAg33.5 has been previously shown to induce immune responses in humans and binds to host erythrocytes [36]. Recently, 10 of 36 PvTRAgs of the Pv-fam-a family were reported to possess erythrocyte-binding activity [35]. Based on transcriptome data, a number of the erythrocyte-binding PvTRAgs including PvTRAg, PvTRAgs, PcTRAg36.6, and PvTRAg69.4 were found in the early stage of the parasite and involve in the rosetting phenomenon [35]; while others including PvTRAg35.2, PvTRAg38, PvTRAg36, and PvTRAg34 were found to express at the late merozoite stage of the parasite that can recognize more than one erythrocyte receptor and help the parasite to invade the host erythrocytes [35]. It appears that the binding of each of the antigens/ligands to different receptors as well as the recognition of each receptor by more than one parasite ligand could be advantageous to *P. vivax*. The parasite can use the redundancy in the receptor-ligand interaction as an alternate invasion pathway or for tightly binding to its host cell during the invasion or rosetting process even in the absence of a Duffy receptor on the erythrocyte surfaces. It merits further investigations on whether the alternate receptor-ligand interactions that allow erythrocyte invasion evolve independently among the *P. vivax* lineages and whether the newly derived *P. vivax* strain has spread among endemic regions subsequent to its emergence.

The geographical distribution of the two *Plasmodium* species varies among sites in Ethiopia. Sites in the southwest (Halaba and Jimma) had a greater proportion of *P. vivax* than *P. falciparum* infections, whereas sites in the north (Mankush and Shewa Robit), with the exception of Bure, had a greater proportion of *P. falciparum* than *P. vivax* infections*.* The predominance of either *Plasmodium* species was reported previously in other parts of the country [23,37-39], and this appears to be dependent on the study population and the season of sampling. Samples of this study were collected from September-November 2013, during the peak season of malaria transmission in Ethiopia. It is unlikely that the difference in the distribution of the two *Plasmodium* species is due to seasonal characteristics of *Plasmodium* infection in Ethiopia, but rather to the variation in climatic conditions among sites [3,40,41]. Despite the fact that the studied area was set in the highlands, sites at lower latitudes (Halaba and Jimma) may experience warmer and more humid weather than sites at higher latitudes (Mankush and Shewa Robit); such climatic variations may influence transmission and distribution of the *Plasmodium* species. Another possible explanation is that difference in the age distribution of the infected individuals may in part influence the distribution of the two *Plasmodium* species. While such an effect is demonstrated by the community samples that indicated a greater prevalence of *P. vivax* infection in children and adolescents than in adults, the prevalence among the clinical samples were not significantly different between the two age groups.

Complicated and severe clinical malaria as well as the prevalence of asymptomatic infections have been previously shown to be highest in young children [42-44]. The acquisition of immunity is age-dependent and young children are most represented among malaria-diagnosed deaths in many African countries [45]. Consistent with previous findings, the present study shows that children aged from 0–5 years old have the highest *P. vivax* (13.9%) and *P. falciparum* prevalence (18.1%) than in adolescents (6.3% and 7%, respectively, for *P. vivax* and *P. falciparum*) and adults (2.6% and 14.2%, respectively, for *P. vivax* and *P. falciparum*). Children and adolescents appear to be more prone to *P. vivax* infection than adults, and the reason for this is not known. One possible explanation is that *P. vivax* can stay dormant in the liver of the adults for a longer time and remain undetected at the time of sample collection. Also, the difference in the level of immunity between children/adolescents and adults may in part influence the invasive capability of the malaria parasite species [45].

## Conclusion

This study provides evidence for *P. vivax* infection in Duffy-negative individuals in Ethiopia and the findings on epidemiology offer evidence-based guidelines for targeting disease control efforts in the most malarious areas of the country.

## Additional files

Additional file 1:
**Number and proportion of** ***Plasmodium vivax*** **and** ***Plasmodium falciparum*** **infections detected in children/adolescents (aged below 18) and adults (aged 18 or above) among the clinical samples collected from the six health centres or hospitals.**
Additional file 2:
**Parasite gene copy number (mean and range values) of** ***Plasmodium vivax*** **and** ***Plasmodium falciparum*** **infections among the clinical samples collected from the six health centres/hospitals across Ethiopia.**
