# Supplementary material for: Molecular epidemiology of Plasmodium vivax and Plasmodium falciparum malaria among Duffy-positive and Duffy-negative populations in Ethiopia
Source: Malar J. 2015 Feb 19;14:84. doi: 10.1186/s12936-015-0596-4 (PMC4340780; doi:10.1186/s12936-015-0596-4)
Supplement: Additional file 1: — Number and proportion of Plasmodium vivax and Plasmodium falciparum infections detected in children/adolescents (aged below 18) and adults (aged 18 or above) among the clinical samples collected from the six health centres or hospitals. [file 12936_2015_596_MOESM1_ESM.docx]

**Additional file 1: Table S1** Number and proportion of *P. vivax* and *P. falciparum* infections detected in children (aged 0-5), adolescents (6-18), and adults (aged above 18) among the clinical samples collected from the six health centers or hospitals.

| Locality |  | Sample size | Number of *P. vivax* infection | Number of *P. falciparum* infection |
| --- | --- | --- | --- | --- |
| Bure |  |  |  |  |
|  | Aged 0-5 | 5 | 5 (100%) | 0 (0%) |
|  | 6-18 | 24 | 15 (62.5%) | 9 (45%) |
|  | >18 | 32 | 17 (53.1%) | 19 (59.4%) |
| Halaba |  |  |  |  |
|  | Aged 0-5 | 7 | 2 (28.6%) | 0 (0%) |
|  | 6-18 | 31 | 3 (9.7%) | 3 (7.9%) |
|  | >18 | 48 | 8 (16.7%) | 2 (4.2%) |
| Jimma |  |  |  |  |
|  | Aged 0-5 | 5 | 3 (60%) | 2 (40%) |
|  | 6-18 | 12 | 5 (41.7%) | 5 (41.7%) |
|  | >18 | 13 | 6 (46.2%) | 5 (38.5%) |
| Mankush |  |  |  |  |
|  | Aged 0-5 | 7 | 0 (0%) | 4 (57.1%) |
|  | 6-18 | 17 | 3 (17.6%) | 14 (82.4%) |
|  | >18 | 26 | 3 (11.5%) | 16 (61.5%) |
| Metehara |  |  |  |  |
|  | Aged 0-5 | 9 | 2 (22.2%) | 8 (88.9%) |
|  | 6-18 | 20 | 8 (40%) | 19 (95%) |
|  | >18 | 24 | 8 (33.3%) | 18 (75%) |
| Showa Robit | |  |  |  |
|  | Aged 0-5 | 5 | 1 (20%) | 4 (80%) |
|  | 6-18 | 15 | 5 (33.3%) | 11 (73.3%) |
|  | >18 | 21 | 5 (23.8%) | 18 (85.7%) |
|  | |  |  |  |
|  | |  |  |  |
| Combined all samples | |  |  |  |
|  | Aged 0-5 | 38 | 13 (34.2%) | 18 (47.4%) |
|  | 6-18 | 119 | 39 (32.8%) | 61 (51.3%) |
|  | >18 | 164 | 47 (28.7%) | 78 (47.6%) |

Note: Not all clinical samples from Jimma were included here because the age data of a subset of samples are unavailable.
