# Supplementary material for: Molecular epidemiology of Plasmodium vivax and Plasmodium falciparum malaria among Duffy-positive and Duffy-negative populations in Ethiopia
Source: Malar J. 2015 Feb 19;14:84. doi: 10.1186/s12936-015-0596-4 (PMC4340780; doi:10.1186/s12936-015-0596-4)
Supplement: Additional file 2: — Parasite gene copy number (mean and range values) of Plasmodium vivax and Plasmodium falciparum infections among the clinical samples collected from the six health centres/hospitals across Ethiopia. [file 12936_2015_596_MOESM2_ESM.docx]

**Additional file 2: Table S2** Log-transformed parasite gene copy number (mean ± standard deviation values) of *P. vivax* and *P. falciparum* infections among the clinical samples collected from the six health centers/hospitals across Ethiopia.

| Locality |  | Parasite species | Sample size | Log-transformed gene copy number/μl |
| --- | --- | --- | --- | --- |
| Bure |  |  |  |  |
|  |  | *P. vivax* | 38 | 3.21±1.11 |
|  |  | *P. falciparum* | 26 | 3.50±1.30 |
| Halaba |  |  |  |  |
|  |  | *P. vivax* | 13 | 2.36±0.91 |
|  |  | *P. falciparum* | 5 | 1.71±1.79 |
| Jimma |  |  |  |  |
|  |  | *P. vivax* | 105 | 2.79±0.83 |
|  |  | *P. falciparum* | 25 | 2.19±1.17 |
| Mankush |  |  |  |  |
|  |  | *P. vivax* | 7 | 2.63±1.91 |
|  |  | *P. falciparum* | 33 | 2.28±0.92 |
| Metehara |  |  |  |  |
|  |  | *P. vivax* | 18 | 2.69±0.84 |
|  |  | *P. falciparum* | 36 | 3.24±1.29 |
| Shewa Robit | |  |  |  |
|  |  | *P. vivax* | 10 | 2.64±1.22 |
|  |  | *P. falciparum* | 33 | 2.99±1.19 |
